# Towards a career in bioinformatics

**DOI:** 10.1186/1471-2105-10-S15-S1

**Published:** 2009-12-03

**Authors:** Shoba Ranganathan

**Affiliations:** 1 Department of Chemistry and Biomolecular Sciences and ARC Centre of Excellence in Bioinformatics, Macquarie University, Sydney NSW 2109, Australia; 2 Department of Biochemistry, Yong Loo Lin School of Medicine, National University of Singapore, 8 Medical Drive, Singapore 117597

## Abstract

The 2009 annual conference of the Asia Pacific Bioinformatics Network (APBioNet), Asia's oldest bioinformatics organisation from 1998, was organized as the 8^th ^International Conference on Bioinformatics (InCoB), Sept. 9-11, 2009 at Biopolis, Singapore. InCoB has actively engaged researchers from the area of life sciences, systems biology and clinicians, to facilitate greater synergy between these groups. To encourage bioinformatics students and new researchers, tutorials and student symposium, the Singapore Symposium on Computational Biology (SYMBIO) were organized, along with the Workshop on Education in Bioinformatics and Computational Biology (WEBCB) and the Clinical Bioinformatics (CBAS) Symposium. However, to many students and young researchers, pursuing a career in a multi-disciplinary area such as bioinformatics poses a Himalayan challenge. A collection to tips is presented here to provide signposts on the road to a career in bioinformatics. An overview of the application of bioinformatics to traditional and emerging areas, published in this supplement, is also presented to provide possible future avenues of bioinformatics investigation. A case study on the application of e-learning tools in undergraduate bioinformatics curriculum provides information on how to go impart targeted education, to sustain bioinformatics in the Asia-Pacific region. The next InCoB is scheduled to be held in Tokyo, Japan, Sept. 26-28, 2010.

## Overview

The Asia-Pacific Bioinformatics Network (APBioNet, [1]) commenced operation in 1998, seeking to encourage scientists from diverse disciplines to work together to address questions in bioinformatics. Following on from annual meetings held in Hawaii, at the Pacific Symposium of Biocomputing (1999-2001), InCoB2002 (the International Conference on Bioinformatics, 2002) in Bangkok, Thailand was adopted as the APBioNet annual conference. Subsequent InCoB meetings were held in Penang, Malaysia (2003); Auckland, New Zealand (2004); Busan, South Korea (2005), New Delhi, India (2006); Hong Kong/Hanoi (by videocasting) (2007) and Taipei, Taiwan (2008).

The second Workshop on Bioinformatics and Computational Biology (WEBCB) was organized at InCoB2009, to facilitate the exchange of curricula, challenges and tools available for bioinformatics education, along with tutorials to train students and new researchers in traditional and emerging topics. The first ever Clinical Bioinformatics Symposium (CBAS) was held prior to the InCoB2009 scientific conference, while the Regional Student Group of the International Society for Computational Biology organized the Singapore Symposium on Computational Biology (SYMBIO) to provide students and young investigators an opportunity to present their ongoing research work.

The scientific meeting featured presentations from articles accepted for peer-reviewed high-impact factor journal publications, in computational biology [1] addressing analysis pertaining to "-omics" data while this supplement features representative bioinformatics research in traditional and emerging areas. Furthermore, a case study on the impact of e-learning tools in bioinformatics education, is presented, to encourage adoption of novel teaching approaches in bioinformatics. Papers submitted to these proceedings were peer-reviewed by at least three reviewers, from the APBioNet/InCoB program committee and invited external experts as required (listed in Additional File 1), with an overall acceptance rate of 50% in BMC journals (details available from [1]).

As a keynote speaker at SYMBIO, I was asked to present a topic of interest to bioinformatics students. Following on from informal advice and mentoring provided over the years, I have attempted to delineate a path for would-be bioinformaticians, embarking on a career in bioinformatics and concluding with a brief description of the diverse areas of bioinformatics research exemplified by articles in this supplement.

## A roadmap for a career in bioinformatics

Domain knowledge is the key to a successful career in bioinformatics. "Computational biology" is not merely a sum of its parts, *viz*. computer science/informatics and biology. It also requires knowledge of mathematics, statistics, biochemistry and sometimes a nodding acquaintance with physics, chemistry and medical sciences. While the list may seem a *pot pourri *of subjects, a student or new researcher needs to equip himself/herself with an in-depth knowledge of the specific problem that is researched and gain essential ingredients of each discipline only to the extent required to address the research questions posed. Becoming an expert on the research topic is the first step on the steep climb to a successful career.

More than a beautiful mind, one must have an inquisitive mind - a veritable thirst for knowledge. To understand a research topic deeply, one must be inclined to ferret out appropriate information and learn new subjects as required, to the extent needed. In a multi-disciplinary area like bioinformatics, a researcher may lose his/her way in the desert sands of scientific literature and books. A careful examination of knowledge sufficiency needs to be exercised here, with a timely return to the research problem itself.

Science is itself a quest for truth and honesty in scientific endeavours is the keystone to a successful career. Scientific integrity in presenting research results and honesty in dealing with colleagues are invaluable to a scientific career, especially one that deals with large datasets. In this context, acknowledging the prior work of other scientists is important.

Communications skills: oral, written and presentation, illuminate the road to success. English is the *lingua franca*, and in the Asia Pacific region, where many scientists are polyglots, command over the English language is essential, for effective communication over e-mail, discussions and question and answer sessions, job and grant interviews, seminars and dissemination of research results as reports and publications. Investing in language training will yield rich dividends. For researchers in bioinformatics, who already possess skills spanning several scientific disciplines, English is just another skill that can be acquired by supervised and unsupervised neural network training. The ability to network and form collaborations also hinges on making comfortable partnerships, stemming from clarity of spoken and written language.

A career is bioinformatics requires problem solving. Here, you need to show persistence in following your hypothesis, even if others think that you are wrong. At the same time, be prepared to modify your hypothesis if the data suggests otherwise. Reaching your ultimate goal is of principal importance, no matter which path you follow.

The art of persuasion is often forgotten in pursuing a bioinformatics career. You must believe in yourself and your work but tactfully convince reviewers and colleagues about your approach, perhaps with the art of suggestion. Without the ability to handle hostile reviewers, the audience or fellow scientists, progress in your career will be slow and difficult. On occasion, rather than a dispute, collaboration may be more beneficial, especially between a bioinformatician and a wet-lab experimental scientist.

Many graduate students simply see their bioinformatics Ph.D. as a goal. For a career, you must make plans for the next year, next three years and maybe even the next five years. Graduate school, your first job, your next job, your publication profile can all be planned as projects using project management tools. Without plans, you are drifting on the internet, without a specific search in mind.

Punctuality or promptness is somehow not given its value. Timelines (again from planning and project management) can assist in setting deadlines for yourself, usually ahead of the real deadline! Labelling tasks as critical, essential and routine can help sort impossible "to-do" lists. These labels need to reviewed periodically and amended as required. You must make the time, for after all, it is relative in Einstein's words and can be stretched.

Critique and criticism are two faces of the same coin. The ability to critically analyse someone else's work such as the assessment of manuscripts and writing reviews can be developed. Here again, communication skills and tact as required. Journal clubs are group opportunities for critique but do not hone the skills of an individual. Reassessing each other's work in a group situation can provide peer-review as well as develop team spirit. More importantly, the ability to take criticism and convert it into something positive is necessary to survive in the hostile world of scientific publication or funding, especially in a multi-disciplinary area such as bioinformatics, where reviewers come from diverse backgrounds and training. To address criticism, emotion must be removed from the equation. Then, address each point carefully and diligently, while sidestepping the harsh language sometimes used. No valid point should be ignored in this exercise. If all else fails, try another avenue of publication or support.

Last but not least, show some initiative and be a pioneer in trying out new ideas or methods. Here, scientific curiosity can suggest new paths. Despite what earlier reports in the literature might support, trust the data and pursue new avenues. Discover new paths (algorithms), new maps (workflows), new places (new data/associations), although when you get there, there is always another challenge ahead.

The stops on this roadmap are only one set of possible paths in a complex network - personalise the points above by adding and deleting your goals. And when you have established a successful career in bioinformatics, remember to help others trudging along.

## Bioinformatics research areas

Among the numerous areas of bioinformatics endeavour, traditional avenues such as sequence analysis, genetic and population analysis, structural bioinformatics, text mining and ontologies are represented in this supplement, while chemoinformatics and biodiversity informatics embody emerging bioinformatics themes. In order to carry out bioinformatics research, innovative teaching is a prerequisite. Improvement in bioinformatics learning is evident from the case study using e-learning tools. Large-scale analysis of "-omics" data have been presented in the InCoB2009 BMC Genomics supplement [1], along with the description of a minimum set of bioinformatics skills in a bioinformatics graduate.

## Sequence analysis

Choo et al. [2] have benchmarked currently available N-terminal signal peptide prediction methods.

## Genetic and population analysis

Kim et al. [3] have developed a genome browser to analyse the variation between the first sequenced Korean genome and other human genomes, to understand predisposition to disease and contribute to preventive health. From entire genomes, Veronika et al. [4] have used a novel bioimaging analysis method to identify populations of cells in a single culture.

## Structural bioinformatics

Lee and Lee [5] describe a new approach to functionally annotate protein sequences by identifying homologous domains, while Dastidar et al. [6] have studied the role of the dynamics of Y100 in the recognition of the tumour suppressor protein p53.

## Text mining and ontologies

Hsu et al. [7] propose a new machine learning approach to identifying abbreviations and definitions in biomedical texts. Applications of text mining include protein subcellular localization prediction [8] and extracting key genes related to specific medical condition [9].

## Emerging areas

Bioinformatics methods are increasingly applied to areas beyond traditional biological data. A chemoinformatic analysis of present day drugs is presented by Khanna and Ranganathan [10] while a biodiversity database on Korean birds is provided by Paik et al. [11].

## Bioinformatics education

Lim et al. [12] share their success of implementing e-learning tools to enhance undergraduate bioinformatics teaching and learning.

## Conclusion

To foster bioinformatics, it is important to actively mentor the next generation of bioinformaticians. The tips provided are by no means comprehensive and will also need to be updated constantly to tackle emerging challenges in mining biological data, be it for genome annotation, personal medicine, drug design or conserving our biodiversity.

## Competing interests

The author declares that they have no competing interests.

## Supplementary Material

Additional file 1 APBioNet InCoB2009 Program Committee members and reviewers. Click here for file
